# Analysis of Theses Written in the Field of Restorative Dentistry in Turkey: A Methodological Study

**DOI:** 10.7759/cureus.91987

**Published:** 2025-09-10

**Authors:** Kemal F Güdül

**Affiliations:** 1 Dentistry, Tokat Gaziosmanpaşa Üniversitesi, Tokat, TUR

**Keywords:** bibliometric analyis, doctorate, restorative dentistry, specialisation, theses

## Abstract

Introduction

This study aims to determine the distribution of theses published in the Department of Restorative Dentistry at the National Thesis Centre of the Council of Higher Education (CoHE) over the last 10 years (2015-2024) according to year, type, university of publication, and field of study through bibliometric analysis, and to evaluate the current status and development of the relevant theses.

Methods

In this study, only theses available to the public on July 20, 2025 at the Turkish National Thesis Centre (www.tez.yok.gov.tr) were evaluated, and no participants were included. PhD and specialization theses completed between 2015 and 2024 in postgraduate education in Restorative Dentistry in Turkey were examined.

Results

Of the total 492 postgraduate theses completed in the field of Restorative Dentistry in Turkey between 2015 and 2024, 64 were PhD theses (13.01%) and 428 were specialization theses (86.99%). The highest number of theses was completed in 2022 (18.7%), while the lowest number was completed in 2015 (2.64%). When examining the universities where the theses were conducted, the highest numbers were at Istanbul University and Cumhuriyet University. A total of 97.15% of the theses were prepared at state universities, while 2.85% were prepared at foundation/private universities. In terms of gender distribution, the percentage of female authors (76.83%) was higher than that of male authors (23.17%). Methodologically, the most frequently preferred type of study was laboratory/in vitro studies (83.13%), while artificial intelligence-based studies were the least preferred method, accounting for only 0.61%.

Conclusions

This study revealed the trends in postgraduate theses in the field of Restorative Dentistry in Turkey between 2015 and 2024. The findings show that some universities stand out in thesis production, female researchers outnumber male researchers, specialization theses dominate over PhD theses, and artificial intelligence-based studies remain quite limited. These data may serve as guidance for future postgraduate education planning and in determining academic priorities.

## Introduction

Postgraduate education is an academic process that follows undergraduate education and includes programmes such as master’s degrees, doctorates (PhD), and artistic proficiency. This process encompasses teaching activities that support theoretical knowledge, scientific research, practical work, and professional development [1].

Restorative dentistry is a branch of dentistry that aims to restore the function, health, and aesthetics of teeth using modern materials and techniques. This treatment seeks to repair and protect teeth damaged by decay, trauma, or developmental disorders [2].

Dentists who wish to pursue postgraduate education in Restorative Dentistry in Turkey can achieve this goal in two ways: first, by applying to PhD programmes, and second, by passing the Specialisation Examination in Dentistry and beginning their specialisation training. Specialist training in Restorative Dentistry involves a structured postgraduate education process lasting three years after undergraduate education. One of the basic elements of this process is thesis work based on scientific research. In order to complete both PhD and specialisation programmes, the thesis must be successfully defended in accordance with the legislation in force [3]. For this reason, the thesis is not only a requirement of postgraduate education but also an indicator of scientific competence.

Specialisation and PhD theses constitute the first and most fundamental step in the scientific research process. The thesis-writing process not only presents postgraduate students with an academic obligation but also provides them with important skills in research methodology. During this process, students acquire essential research skills such as defining a scientific problem, developing hypotheses, applying appropriate data collection methods, analysing the data obtained, and interpreting the findings within a scientific framework. Thus, thesis work is an important educational tool for developing an individual’s identity as a researcher [4].

Bibliometric analysis has become one of the most widely used methodological approaches in academic studies in recent years. Bibliometrics, as an applied method that enables the systematic analysis of extensive scientific data, makes significant contributions to the quantitative assessment of scientific productivity, impact levels, and research trends. In this regard, bibliometric analysis is an effective tool not only for revealing the structural characteristics of scientific publications but also for understanding developments and trends in research fields [5].

To date, there are no studies on theses completed at the doctoral or specialist level in Restorative Dentistry. The primary objective of this study is to conduct a comprehensive bibliometric analysis of postgraduate theses prepared in the field of Restorative Dentistry in Turkey over the past decade (2015-2024). The study examined doctoral and specialist theses available at the National Thesis Centre and evaluated their distribution by year, thesis type (doctoral/specialist), author gender, university, and study type. In this way, the current state and development trends of academic production in the field of restorative dental treatment were revealed, with the aim of providing guiding data for future postgraduate education planning and research priorities.

## Materials and methods

Data collection

In this study, publicly available and freely accessible postgraduate theses in the Turkish Higher Education Council's National Thesis Database were examined. As the research had no direct impact on humans or animals, ethical committee approval was not required. The study was conducted in accordance with the principles of the Helsinki Declaration. In the thesis search, only theses available to the public as of July 20, 2025 on the Turkish National Thesis Centre (www.tez.yok.gov.tr) were evaluated, and no participants were included in the study. Doctoral and specialisation theses completed in postgraduate education in Restorative Dentistry in Turkey were examined. A search was conducted using the advanced search function to determine which theses would be included. The search was filtered according to the field of study to cover the years 2015-2024, and only doctoral and specialisation theses in the field of Restorative Dentistry were retrieved. Online access was provided to a total of 492 postgraduate theses. To determine the types of theses examined within the scope of the study, each thesis file was downloaded in PDF format from the National Thesis Centre, and the abstract and materials and methods sections were subjected to detailed analysis.

Eligibility criteria

The inclusion criteria were as follows: doctoral or specialist theses in the field of Restorative Dentistry that had been conducted and completed at state or foundation/private universities in Turkey, uploaded to the National Thesis Centre, and completed between 2015 and 2024. The starting year of 2015 was chosen because the first final theses prepared as part of specialist training in dentistry were completed in that year. The year 2025 was excluded as it had not yet been completed. The exclusion criteria were: theses uploaded to the National Thesis Centre but not yet completed, master’s theses, theses outside the 2015-2024 period, and theses outside the field of Restorative Dentistry. The theses included in the study were evaluated under the following headings: year, university name, university type (state or foundation/private), thesis type (doctoral or specialisation), author’s gender, number of theses completed at universities, and methodology.

Statistical analysis

The data obtained in the study were transferred to Microsoft Excel and grouped according to various criteria. In this context, the distribution of theses by year, university, university type (public/foundation), thesis type (doctoral/specialisation), author gender, and study type (in vitro, in vivo, retrospective, survey, AI, etc.) was evaluated. Basic statistical methods (totals and percentages) were applied, and frequency tables and graphs based on these tables were prepared. In this way, the current status of thesis studies conducted in the field of Restorative Dentistry between 2015 and 2024 was systematically mapped.

## Results

Between 2015 and 2024, a total of 492 theses were completed, including 64 PhD theses and 428 specialisation theses. The highest number of theses was completed in 2022 (92 theses, 18.7%), while the lowest number was in 2015 (13 theses, 2.64%). The distribution of theses by year is presented in Table 1.

**Table 1:** Distribution of theses by year.

Year	Number of Theses	Percentage (%)
2015	13	2.64
2016	24	4.88
2017	39	7.93
2018	36	7.32
2019	49	9.96
2020	48	9.76
2021	59	11.99
2022	92	18.7
2023	78	15.85
2024	54	10.98

When examining the distribution of theses according to the universities where they were conducted, thesis records from a total of 41 universities were accessed. The universities with the highest number of completed theses were Istanbul University and Cumhuriyet University (30 theses, 6.1%) among state universities, and Bezm-i Alem University (11 theses, 2.24%) among foundation/private universities (Table 2).

**Table 2:** Distribution of theses by university.

University	Number of Theses	Percentage (%)
İstanbul / Cumhuriyet	30	6.1
Selçuk	28	5.69
Ondokuz Mayıs	26	5.28
Marmara	25	5.08
Atatürk	24	4.88
Gazi / Dicle	23	4.67
Akdeniz	21	4.27
Kırıkkale	20	4.07
Ege	19	3.86
Kocaeli / Hacettepe	18	3.66
Karadeniz Teknik	17	3.46
Erciyes / İzmir Katip Çelebi	16	3.25
Gaziantep	13	2.64
Gaziosmanpaşa / Sağlık Bilimleri	12	2.44
Eskişehir Osmangazi / Recep Tayyip Erdoğan / Necmettin Erbakan / Bezm-i Alem / Süleyman Demirel	11	2.24
Abant İzzet Baysal / Ankara / Ordu	7	1.42
Hatay Mustafa Kemal / Aydın Adnan Menderes	6	1.22
İnönü / Yeditepe / Yüzüncü Yıl / Başkent	5	1.02
Pamukkale	4	0.81
İstanbul Medipol	3	0.61
Afyonkarahisar Sağlık Bilimleri / Trakya / Afyon Kocatepe	2	0.41

When examining whether the universities were state or foundation/private, 478 theses (97.15%) were completed at state universities and 14 theses (2.85%) at foundation/private universities (Table 3).

**Table 3:** Distribution of theses by university type.

University Type	Number of Theses	Percentage (%)
State	478	97.15
Private / Foundation	14	2.85

When the distribution of theses by type was examined, 64 were PhD theses (13.01%) and 428 were specialisation theses (86.99%) (Table 4).

**Table 4:** Distribution of theses by thesis type.

Thesis Type	Number of Theses	Percentage (%)
PhD	64	13.01
Specialisation	428	86.99

When the distribution of theses according to the gender of the author was examined, 378 theses (76.83%) were written by female authors, while 114 theses (23.17%) were written by male authors (Table 5).

**Table 5:** Distribution of theses by author gender.

Year	2015	2016	2017	2018	2019	2020	2021	2022	2023	2024	Total
Male	7	8	7	14	10	11	11	14	18	14	114
Female	6	16	32	22	39	37	48	78	60	40	378

When examining the distribution of theses according to study type, the most commonly used type was laboratory/in vitro studies (409 theses, 83.13%), while the least frequently used was retrospective studies (8 theses, 1.63%) (Table 6).

**Table 6:** Distribution of theses by study type.

Study Type	Number of Theses	Percentage (%)
Laboratory work / in vitro	409	83.13
Clinical study / in vivo	50	10.16
Survey	22	4.47
Retrospective	8	1.63

## Discussion

A thesis is an academic work that reports on scientific research conducted in a systematic, impartial, and theoretically based manner, focusing on a specific topic [6]. It is also one of the basic components of both PhD and specialisation programmes. Analysing trends in this important stage of postgraduate education not only provides a general assessment of the academic approach in the field of Restorative Dentistry in our country but also serves as a valuable guide for researchers in directing their future theses and article studies.

Various bibliometric studies have been conducted to evaluate publication trends in restorative dentistry across different journals and time periods. However, the findings of these studies are generally not comparable, as the parameters, journals, and time periods evaluated are highly diverse. Most of these bibliometric studies have focused solely on popular orthodontic journals or specific parameters such as subject or study design type, while others have evaluated articles with a high number of citations. These studies have generally concentrated on either article-related factors or design- and author-related factors; studies examining both are rare [7, 8]. Our study was conducted to evaluate both the methodology of the theses and the characteristics of the researchers.

Özkalaycı N et al. reported that specialisation training in dentistry was preferred over PhD programmes [9]. Similarly, in this study, the number of specialisation theses in dentistry between 2015 and 2024 was significantly higher than the number of doctoral theses. This trend is thought to stem from the growing interest in specialisation training and the increase in the number of available places in both specialisation and PhD programmes at newly established dental faculties. The professional status and economic advantages that the title of specialist provides to dentists are considered to play a decisive role in the preference for specialisation training over PhD programmes.

Güler Ç and Alper ES reported that the number of female authors was higher than that of male authors in their study examining master’s theses in paediatric dentistry between 2012 and 2022 [10]. Meriç P and Kılınç DD likewise reported that female authors outnumbered male authors in their study examining postgraduate theses in orthodontics between 2017 and 2021 [11]. Similarly, the present study found that female authors wrote a higher number of theses in the field of Restorative Dentistry than male authors. Based on these findings, it can be said that postgraduate education in Restorative Dentistry is more popular among female dentists and that this field has increasingly attracted female researchers.

Meriç P and Kılınç DD reported in their study in the field of orthodontics that Süleyman Demirel University had the highest number of completed theses [11]. Güler Ç and Alper ES reported in their study in the field of paedodontics that Marmara University had the highest number of completed theses [10]. Based on the findings of this study, the highest number of published theses were produced at Istanbul University and Cumhuriyet University. It is believed that factors such as these institutions having a long-standing academic history, being located in large cities and therefore being preferred by students, and employing a large number of experienced faculty members played a role in the high number of postgraduate theses at these universities. Furthermore, when examining the total number of postgraduate theses completed by universities between 2015 and 2024, it was observed that no foundation (private) universities were among the top ten. When the overall picture is evaluated, it is striking that the number of theses produced by private universities is significantly lower than that of state universities. One of the main reasons for this is that the vast majority of private universities are relatively new and have therefore not yet developed a regular, sustainable academic publication and research culture. In addition, the fact that private universities are mostly income-oriented, while state universities focus more on academic production and research, can also be considered among the important factors explaining this difference in total thesis production [12, 13].

In their study, Meriç P and Kılınç DD reported that, when examined according to methodology, more clinical studies (in vivo) were conducted in the field of orthodontics [11]. Güler Ç and Alper ES reported that, when examined according to methodology, more laboratory studies (in vitro) were conducted in the field of paedodontics [10]. However, in our study, when we examined the methodology of theses completed in the field of Restorative Dentistry, we found that laboratory work (in vitro) was more common. This can be explained by differences in clinical work in specialisation areas, the long duration of clinical studies, ethical concerns, and the fact that specialisation training in Restorative Dentistry is shorter than in some other branches, although rotation training is more extensive.

In addition to limitations related to data availability, this study has several other limitations. First, the fact that the data were obtained only from specific institutions and centres may have introduced institutional bias into the results. In addition, there is a risk of incorrect or incomplete coding in the classification of variables such as methodology type or gender. Furthermore, the fact that the content of the theses was not evaluated qualitatively has limited the study to quantitative data only. These factors should be considered when interpreting and generalising the findings. The lack of similar studies in the field also limits the comparative evaluation of the results. 

This study included only doctoral and specialisation theses completed between 2015 and 2024; theses prepared in 2025 were excluded. As a result, the most recent theses were not included in the analysis, and the latest trends in the field may not be fully reflected. This limitation should also be taken into account when interpreting the findings. Another limitation is that it was not possible to access theses that had not been uploaded to the Higher Education Council National Thesis Centre database. More comprehensive and multi-centre studies are needed to gain a clearer understanding of academic orientations in the field of Restorative Dentistry.

## Conclusions

This study presents, to the best of our knowledge, the first comprehensive analysis of doctoral and specialist theses in the field of Restorative Dentistry in Turkey. By examining postgraduate theses completed between 2015 and 2024, it reveals the dominant trends and characteristics of scientific productivity in this discipline. The findings show that certain universities and female researchers stand out, that specialist theses outnumber doctoral theses, that state universities are more productive than foundation/private universities, and that AI-based studies remain limited in number. These results emphasize the need for strategic planning in shaping future graduate education policies, academic orientations, and research priorities. More comprehensive and multi-centre studies should be conducted in the field to enable a more detailed assessment of the evolution of these academic trends over time.
